# Human Coronavirus Spike Protein Based Multi-Epitope Vaccine against COVID-19 and Potential Future Zoonotic Coronaviruses by Using Immunoinformatic Approaches

**DOI:** 10.3390/vaccines10071150

**Published:** 2022-07-19

**Authors:** Zulqarnain Baloch, Aqsa Ikram, Saba Shamim, Ayesha Obaid, Faryal Mehwish Awan, Anam Naz, Bisma Rauff, Khadija Gilani, Javed Anver Qureshi

**Affiliations:** 1 Faculty of Life Science and Technology, Kunming University of Science and Technology, Kunming 650500, China; znbalooch@yahoo.com; 2 Institute of Molecular Biology and Biotechnology (IMBB), University of Lahore (UOL), Lahore 54000, Pakistan; saba.shamim@imbb.uol.edu.pk (S.S.); anam.naz@imbb.uol.edu.pk (A.N.); khadija.gilani@imbb.uol.edu.pk (K.G.); javed.anver@imbb.uol.edu.pk (J.A.Q.); 3 Department of Medical Lab Technology, University of Haripur (UOH), Haripur 22620, Pakistan; ayesha_obaid_nust@live.com (A.O.); faryal_mehwish@yahoo.com (F.M.A.); 4 Department of Biomedical Engineering, University of Engineering and Technology (UET) Lahore, Narowal Campus, Narowal 51601, Pakistan; bisma.rauff@uet.edu.pk

**Keywords:** SARS-CoV-2, bat SL-CoV and SARS-CoV, immunoinformatics, vaccine

## Abstract

Zoonotic coronaviruses (CoV) have emerged twice and have caused severe respiratory diseases in humans. Due to the frequent outbreaks of different human coronaviruses (HCoVs), the development of a pan-HCoV vaccine is of great importance. Various conserved epitopes shared by HCoVs are reported to induce cross-reactive T-cell responses. Therefore, this study aimed to design a multi-epitope vaccine, targeting the HCoV spike protein. Genetic analysis revealed that the spike region is highly conserved among SARS-CoV-2, bat SL-CoV, and SARS-CoV. By employing the immunoinformatic approach, we prioritized 20 MHC I and 10 MHCII conserved epitopes to design a multi-epitope vaccine. This vaccine candidate is anticipated to strongly elicit both humoral and cell-mediated immune responses. These results warrant further development of this vaccine into real-world application.

## 1. Introduction

Coronaviruses are enveloped, single positive-stranded RNA viruses that have been discovered in a wide spectrum of hosts including humans, bats, camels, mice, and hedgehogs. These viruses belong to the family *Coronaviridae* and subfamily *Coronavirinae*, which are further subdivided into four groups, the alpha, beta, gamma, and delta coronaviruses [1]. In general, coronavirus infection in humans is self-limiting and only causes mild symptoms associated with the common cold. A new member of coronaviruses was identified in late December 2019 and designated as SARS-CoV-2. It was epidemiologically linked to a wet market in Wuhan, China, and is considered of zoonotic origin [2]. As of 22 February 2022 (according to Chinese CDC; 2019ncov.chinacdc.cn/2019-nCoV (accessed on 22 February 2022), more than 76,000 cases of COVID-19 have been confirmed in China. Similar to the previously identified SARS-CoV and MERS-CoV, SARS-CoV-2 causes severe acute respiratory diseases. However, it is more contagious with the capability of human-to-human transmission but is less pathogenic with a mortality rate of approximately 2–3%. Genetically, it is closely related to SARS-CoV and bat SARS-like coronavirus (SL-CoV), but more distal to MERS-CoV [3]. 

SARS-CoV-2 shows similarities with the genome composition of SARS-CoV, MERS-CoV, and other human coronaviruses. The 3′-terminus of the coronavirus genome encodes four major structural proteins including spike (S), envelope (E), membrane (M), and nucleocapsid (N) proteins. The 3′-terminus of the genome also encodes multiple accessory proteins that are usually genus-specific and helps to increase the virulence or evade immune response [4]. For instance, SARS-CoV contains accessory protein ORF 3a, 3b, 6, 7a, 7b, 8a, 8b and 9b; MERS-CoV contains ORF 3, 4a, 4b, 5, 8b; and SARS-CoV-2 contains ORF 3a, 6, 7a, 7b, 8, 10. Although there have been numerous attempts to develop vaccines against HCoV infections in recent decades, their considerable sequence diversity is a limiting factor. Given the high homology between SARS-CoV-2 and other coronaviruses, there is a high possibility of conserved antigenic epitopes [5]. Therefore, previous exposure to MERS, SARS, or seasonal human coronaviruses (i.e., 229E, NL63, OC43, and HKU1) may contribute to modulating immunity against SARS-CoV-2 infection.

**Figure 1:**
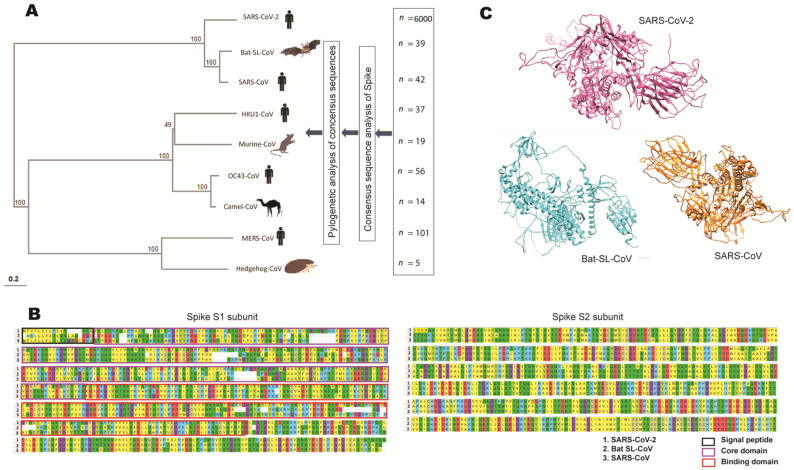
(**A**) The phylogenetic analysis of the spike consensus sequences of SARS-CoV-2 and other representatives of the genus Betacoronavirus. The consensus sequences were generated from genome sequences of SARS-CoV-2 (*n* = 6000), HKU1-CoV (*n* = 37), SARS-CoV (*n* = 42), MERS-CoV (*n* = 101), OC43-CoV (*n* = 56), bat-SL-CoV (*n* = 39), murine-CoV (*n* = 19), camel-CoV (*n* = 14), and hedgehog-CoV (*n* = 5) [10,11]. (**B**) A comparison of the protein sequences of the S1 and S2 subunits of the spike region with the consensus sequences of SARS-CoV-2, bat SL-CoV, and SARS-CoV. Analysis was performed and displayed using MEGAX. (**C**) Tertiary structures of the spike region based on the consensus sequences of SARS-CoV-2, bat SL-CoV, and SARS-CoV predicted by ITASSER. CoV: coronavirus; MERS: Middle East respiratory syndrome; SARS: severe acute respiratory syndrome; SL: SARS-like.

No common effective pan-CoV licensed vaccines are available yet that have broad activity against these zoonotic viruses. Currently, scientists and biotech industries are in a race to develop a vaccine against SARS-CoV-2. However, conventional approaches require a yearlong process to develop a vaccine with the major issue of containment including biosafety and biosecurity requirements. This limitation can be overcome by using immunoinformatic approaches. Targeted delivery of vaccine antigen to antigen-presenting cells (APCs) is a major goal of immunoinformatic based vaccines [6]. This enhances vaccine exhibition and is an effective strategy for making more efficient vaccines. The induction of robust T-cell responses is required to increase the relevance for vaccine efficacy [7]. In a multiepitope vaccine processing pathway, the exogenous vaccine antigens are internalized by endosome, cleaved by proteases, presented to MHCII molecules in endosomes, and transported to the cell membrane for the priming of CD4+ T-cells. There are two pathways for cross-presentation of the multiepitope vaccine candidate. In the cytosolic pathway, exogenous vaccine antigens are endocytosed and chopped by proteases. The resulting peptides either escape by the endosomal membrane via endoplasmic reticulum associated degradation (ERAD). The escaped peptides will be processed by the proteasome and transported to ER where they are packaged onto MHC-I with the help of TAP and finally transported onto the cell membrane for CD8+ T-cell activation. These peptides can also be digested by the proteasome and transported back to the endosomes where they are loaded onto MHC-I molecules and subsequently transported to the cell membrane for CD8+ T-cell priming. In the vacuolar pathway, in contrast, the antigens will be degraded inside the endosome and directly loaded onto the MHC-I molecules that will be transported onto the cell membrane for CD8+ T-cell stimulation (Figure 1). In the classical MHC-I pathway, the proteasome will digest the endogenous vaccine antigens in the cytosol and transport it to the endoplasmic reticulum (ER) for loading onto MHC-I molecules with the help of the TAP protein. Here, the MHC-I-peptide will be transported onto the cell membrane for the CD8+ T cell priming [8].

In the current study, we targeted the spike (S) glycoprotein of the coronaviruses for the prediction of a pan-CoV vaccine. The glycoprotein of the coronavirus consists of two subunits, S1 and S2. It is responsible for recognizing the host cell receptors and is considered as a major target for coronavirus vaccine development [9]. By employing immunoinformatic approaches, we first analyzed the evolutionary features and genetic conservation of the spike (S) region of the coronavirus followed by mapping of the immunogenicity and epitope profile of the spike protein. We finally demonstrate a proof-of-concept for designing a multi-epitope vaccine against SARS-CoV-2 that can prove useful from a post-pandemic perspective to prevent subsequent infectious waves.

## 2. Materials and Methods

### 2.1. Sequence Retrieval

The genome sequences of SARS-CoV-2 (*n* = 6000), HKU1-CoV (*n* = 37), SARS-CoV (*n* = 42), MERS-CoV (*n* = 101), OC43-CoV (*n* = 56), bat-SL-CoV (*n* = 39), murine-CoV (*n* = 19), camel-CoV (*n* = 14), and hedgehog-CoV (*n* = 5) were retrieved from Genbank [10] and the virus pathogen resource database (ViPR) [11]. GenBank (http://www.ncbi.nlm.nih.gov (accessed on 23 February 2022) is a comprehensive database that contains publicly available nucleotide sequences obtained primarily through the sequence data received from individual laboratories and large-scale sequencing projects [12]. The workflow of the ViPR database provides access to sequence records, immune epitopes, 3D structures, gene and protein annotations as well as the host factor data of a broad range of virus pathogens including the entire *Coronaviridae* family [13].

### 2.2. Sequence Conservation, Similarity and Phylogenetic Analysis

In order to find the conserved regions of selected coronavirus sequences, sequence alignment was conducted. Sequences of the coronavirus spike protein from all groups were aligned by using CLC workbench7 [14], MEGAX [15], and Bioedit software [16] to generate the consensus sequence. All of the selected sequences with a similarity greater than 50% in relation to SARS-CoV-2 were subjected to further analysis. Overall conserved regions (OCR) of these species (similarity > 50%) were obtained by aligning their consensus sequence. Blastp [17] was used to calculate the similarity percentage with the human proteome to avoid an autoimmune response. Phylogenetic analyses of the spike regions were performed using RAxML software (version 8.2.9) [18] with 1000 bootstrap replicates by employing the general time reversible nucleotide substitution model.

### 2.3. Epitope Mapping and Selection

For the production of an epitope-based vaccine, T-cell (MHC-I and MHC-II) epitope prediction is very important. By using a consensus sequence of the selected coronavirus spike, MHC I and MHC II epitopes were predicted by the HLApred server [19] and by an online immune epitope database resource (IEDB) available online http://tools.iedb.org/bcell/ (accessed on 25 March 2022) [20]. IEDB, Discotope (http://www.cbs.dtu.dk/services/DiscoTope (accessed on 25 March 2022)) and Ellipro [3] software were also used for B-cell epitope prediction. Epitopes predicted by more than one software were considered for further analysis. In order to select suitable B-cell epitopes, the prediction was conducted on the basis of their antigenicity, surface accessibility, flexibility, beta turn, and hydrophilicity. Only epitopes that were present on the outer surface were selected. Intracellular epitopes were eliminated. In order to determine the IFN-gamma inducing MHC class II binding peptides, the IFN epitope server was used [6]. Furthermore, all of the T-cell epitopes overlapped with the B-cell and IFN-γ epitopes, and those also present in conserved regions were prioritized. The antigenicity of the selected epitopes was analyzed by using the Vaxijen server [21]. The epitopes with higher antigenicity were selected for further analysis. To construct a sub-unit vaccine, the epitopes with the following properties were selected: (i) antigenic, (ii) conserved, (iii) overlapping with B and IFny epitopes, (iv) having high binding affinity with human allele, and (vi) have no similarity to the human proteins. 

### 2.4. Multi-Epitope Vaccine Design and Secondary/Tertiary Structure Prediction

All of the selected epitopes were linked together with the AAY linker. AAY linkers increase the structural stability of epitopes by avoiding self-folding, improve the epitope presentation, and enhance the immunogenicity of th vaccine construct [22]. To further increase the immunogenicity of the vaccine construct, β-defensin (45 amino acids; GIINTLQKYYCRVRGGRCAVLSCLPKEEQIGKCSTRGRKCCRRKK) was added at its N-terminal by the EAAAK linker. The 3D structure of the vaccine construct was predicted by I-TASSER [23]. I-TASSER is an integrated server and a hierarchical protein structure modeling approach based on the sequence-to-structure-to-function paradigm and structure convergence of the Monte Carlo simulations [24]. A higher C-score of the predicted structure represents the model with a higher stability and confidence. A further secondary structure was evaluated by PSIPRED [25]. Refinement of the predicted structure was conducted by using GlaxyRefine (http://galaxy.seoklab.org/cgi-bin/submit.cgi?type=REFINE (accessed on 28 March 2022)) and Modrefiner [26]. These two methods can improve the quality of the vaccine construct. GalaxyRefine server performed the best in improving the local structure quality according to community-wide CASP10 experiments [27]. Structure validation was performed by the Ramachandran plot (http://mordred.bioc.cam.ac.uk/~rapper/rampage.php accessed on 3 April 2022) and PROSA server [28].

### 2.5. Comparison of Secondary Structures

The secondary structures of the selected spike protein were predicted by using PSIPRED [25] and PDBsum [29]. The online server PRISPRED (http://bioinf.cs.ucl.ac.uk/psipred (accessed on 6 April 2022), efficiently generates the secondary structure as well as predicts the transmembrane topology, transmembrane helix, fold, and domain recognition regions of a given query protein. The PDBsum database portrays the structural features and molecular composition of DNA, ligands, proteins, and metal ions as well as a pictorial representation of their interactions [30]. These structures were evaluated for changes in the alpha helix, strands, and coils in spike proteins.

### 2.6. Evaluation of Physiochemical Properties and Molecular Docking of the Vaccine Construct with Toll-like Receptor 3

The physical and chemical characteristics of the final vaccine construct including molecular weight, aliphatic index, grand average of hydropathicity, theoretical isoelectric point, in-vitro and in-vivo half-life and stability index were evaluated by using online server Protparam (http://web.expasy.org/protparam (accessed on 6 April 2022). Molecular docking analysis of the vaccine construct with Toll-like receptor (TLR3) was performed by information-driven, integrative flexible docking with the HADDOCK server [31]. HADDOCK makes use of a variety of restraints including NMR chemical shift perturbations, chemical cross-linking, hydrogen/deuterium exchange, and mutagenesis during the docking process to drive and score the complex formation [32].

## 3. Results

### 3.1. The Evolutionary Features SARS-CoV-2 Spike Region

Phylogenetic analysis is used to evaluate the evolutionary relatedness among different viruses by using their genomic sequences. To comprehensive map and avoid sequence selection bias, we first retrieved a large number of spike sequences from different types of coronaviruses (human and zoonotic). We then performed consensus sequence analysis to generate one consensus sequence for each type (Supplementary Figure S1). These sequences were subjected to phylogenetic analysis. As shown in the phylogenetic tree, the spike of SARS-CoV-2 is closely related to bat SL-CoV and SARS-CoV, but distal to other members KU1-CoV, MERS-CoV, OC43-CoV, camel-CoV, murine-CoV, and hedgehog-CoV (Figure 1A). The betacoronaviruses that are important to human health include OC43, HKU1, SARS-CoV, SARS-CoV-2, and MERS-CoV. The natural reservoir for betacoronaviruses are bats and rodents. Therefore, in the present study, we used the spike protein of the human infecting coronavirus and the rodent infecting betacoronavirus.

### 3.2. Genetic Conservation Analysis

Blastp analysis of the consensus sequence of the SARS-CoV-2 spike region showed a 77% identity with bat SL-CoV and 76% identity with SARS-CoV. Additionally, no identity was observed with the human proteome. The conserved regions greater than nine amino acids were observed with these two betacoronavirus. Within the S1 subunit of the spike, the signal peptide of SARS-CoV was only 21% identical to bat SL-CoV and 58% identical to SARS-CoV. The core domain was 56% identical to bat SL-CoV and 52% to SARS-CoV. The binding domain had a 71% similarity with bat SL-COV and 73.6% with SARS-CoV (Figure 1B). The S2 domain was highly conserved and showed a 92% similarity with bat SL-CoV and 90% with SARS-CoV (Figure 1B). Thus, the S2 domain represents an ideal target for vaccine and drug design. Overall, 27 conserved regions (>9 amino acids) were identified among the spike regions of these three types of coronaviruses (Supplementary Table S1). In contrast, much less similarity of the SARS-CoV-2 spike to KU1-CoV (35%), MERS-CoV (35%), OC43-CoV (38%), camel-CoV (38%), murine-CoV (36.4%), and hedgehog-CoV (33.5%) was observed, and no conserved region greater than nine amino acids was identified.

### 3.3. Epitope Mapping of Spike Protein

Based on the analyzed genetic features, we believe that the spike is a candidate target for multi-epitope vaccine development. We thus adopted strict and regressive criteria to identify the epitopes. Nine mer MHC I and II epitopes with a binding affinity to the maximum number of alleles were predicted against the spike region of SARS-CoV-2, bat SL-CoV, and SARS-CoV. Furthermore, twenty mer B-cell and nine mer IFN-γ epitopes were also predicted. All T-cell epitopes overlapping the B-cell and IFN-γ epitopes and present in the conserved regions based on the consensus sequences were screened out. By evaluating the antigenicity, epitopes with an antigenicity less than 5 threshold were excluded for further study. Blastp was performed to avoid any human protein homologous epitope and autoimmune response. Based on the strict screening approach, the prioritized epitopes had the following features: (1) Maximum binding affinity to a large number of human alleles (2) present in the conserved region and colocalized with predicted epitopes (IFN-γ and B-cell), and (3) highly antigenic and nonhuman homologs. Based on these filters. Based on these criteria, we finally prioritized 20 MHC I (named T1-T20) and 10 MHCII epitopes (named L1-L10) for the vaccine design (Table 1).

We next characterized the secondary (Supplementary Figure S1) and tertiary structures of the spike (Figure 1C) based on the consensus sequences of SARS-CoV-2, bat SL-CoV, and SARS-CoV.

### 3.4. Multi-Epitope Vaccine Design and Characterization

**Figure 2:**
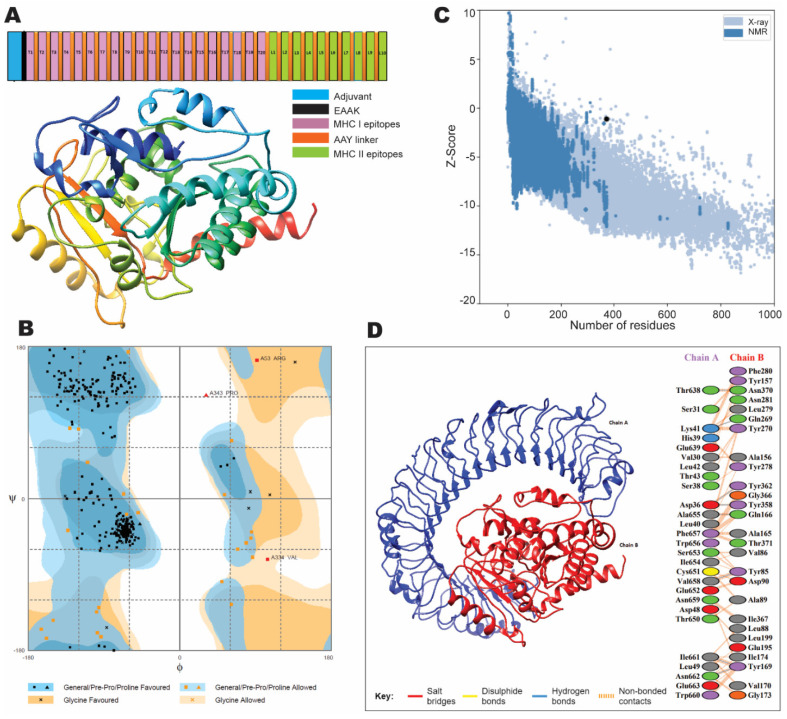
(**A**) A schematic illustration of the vaccine construct showing epitopes with linkers and adjuvant (β-defensin). The predicted tertiary structure (3D) of the vaccine construct by homology modeling on I-TASSER. (**B**) Validation of the refined tertiary structure with Ramachandran plot analysis showing 92.7% region in favored region, 6.5% in allowed regions, and 0.8% in outlier regions. (**C**) The tertiary structure validation by ProSA-web showing a Z-score of −1.5. (**D**) Molecular docking of the vaccine construct with the immune receptor TLR3. Red color indicates the vaccine construct (chain B) while the blue color indicates TLR3 (chain A). Molecular interactions are shown between chain A and B.

To design a multi-epitope vaccine, the prioritized epitopes were joined together by using the AAY linker. The AAY linker increases the structural stability and immunogenicity of the vaccine construct. An adjuvant β-defensin was added at the N-terminal of the vaccine construct by the EAAAK linker to enhance the antigenicity (Figure 2A). Overall antigenicity was confirmed by analysis with the Vaxijen server, and allergenicity was checked by AllergenPro, confirming that it was not an allergen in nature. Overall antigenicity of the vaccine construct was 2, proposing the vaccine construct as antigenic in nature.

### 3.5. Secondary and Tertiary Structure Prediction and Validation

The information regarding the quality of the secondary and tertiary structure of the vaccine construct is of significant importance in designing a vaccine. It is important for the efficient presentation of epitopes on MHC to stimulate a strong immune response [33,34]. The secondary structure of the vaccine was predicted by PESIPRED and PDBsum. The secondary structure analysis showed that it had three sheets, three beta alpha beta units, two beta hairpin, one beta bulge, nine strands, 18 helices, 14 helix–helix interacts, 37 beta turns, and 13 gamma turns (Supplementary Figure S2). The further tertiary structure was predicted by using I-TASSER [35]. Five 3D structures of vaccine construct were predicted by the I-TASSER on the basis of ten threading templates, with confidence score (C-score) values (−3.96 to −1.31). Usually, the high C score model represents high stability and confidence. Tertiary structure refinement (Figure 2A) indicates that an area of 92.7% of the vaccine construct was in the favorable region, 6.5% in the allowed region while 0.8% was in the outlier region, suggesting that it is a stable structure (Figure 2B). The predicted model quality was further confirmed by ProSA, showing a Z score as 1.5 (Figure 2C).

### 3.6. Prediction of Physiochemical Properties

The ideal vaccine construct should have viable physiochemical properties. The physiochemical properties of this vaccine candidate were estimated by ProtParam. The molecular weight of the vaccine construct was 40,812 and the isoelectric point was 8.5, thus representing it as basic in nature. The vaccine half-life was estimated to be 30 h in mammalian reticulocytes in vitro, less than 20 h in yeast in vivo, and less than 10 h in Escherichia coli in vivo. The instability index was calculated to be 26.91 classifying it as a stable protein. The aliphatic index was 94.04 and the grand average of hydropathicity was 0.134.

### 3.7. Molecular Docking Analysis with TLR3

The vaccine candidate association with a specific immune receptor is required in the activation of innate immunity and the synchronization of the adaptive immune response after the recognition of pathogen associated molecular patterns (PAMPs) of pathogens. The immune receptor TLR3 is essential in defending coronavirus infections [36]. Interestingly, molecular docking indicates active interactions between our vaccine construct with TLR3. The vaccine construct developed eight hydrogen bonds with TLR3. All of the hydrogen bonds were laid at a distance of 2–3 Å, revealing strong interactions (Figure 2D). These results collectively suggest that this designed vaccine is stable and highly immunogenic, holding promise for further development.

## 4. Discussion

The prevalence of SARS-CoV-2 has raised concerns regarding future pandemics. Therefore, there is an urgent need to develop pan-coronavirus vaccines that can target not only the current SARS-CoV-2 variants of concern, but also future putative pandemics. Although dozens of ‘universal’ coronavirus vaccines are in development, there is no pan-coronavirus vaccine. Therefore, there is an urgent need for a pan-coronavirus vaccine with broad neutralizing activity. Powerful genomics technologies have a significant impact on reverse vaccinology, and decreased the time for vaccine development. Genome mining’, and the information driven from functional and structural proteomics, provide novel approaches for a more rapid screening of antigens, leading to a third generation of vaccines. In addition, pan-genomic reverse vaccinology, with the comparison of protein sequences from various isolates of the same species of a pathogen, increases the possibility of the identification of conserved vaccine candidates [37]. Based on these technologies, immunoinformatic approaches can be applied to design a pan-coronavirus vaccine.

The coronavirus spike protein is the major surface protein that binds to the host cell receptor and mediates virus entry. Thus, the genetic information within the spike region constitutes the essential clues for understanding virus evolution, transmission, and pathogenesis. The receptor binding domain in the spike protein is the most variable part of the virus genome. This region of SARS-CoV-2 is also closely related to bat SL-CoV and SARS-CoV [38]. Instead of using individual coronavirus sequences, we mapped the genetic features of the spike by generating the consensus sequences for each type of coronavirus from a large set of individual sequences. Such information is important when considering sequence-dependent functions of the viral proteins. Our results confirmed that the spike of SARS-CoV-2 is most closely related to bat SL-CoV and SARS-CoV. Importantly, our conservation analysis has paved the way for the design of a multi-epitope vaccine targeting the spike protein. In this study, we took the advantage of an immunoinformatics strategy, and designed a multi-epitope vaccine based on highly conserved regions of the spike protein. These selected epitopes have the potential to generate more specific, effective, strong, and long-lasting immune responses while avoiding undesired side effects. This pan-coronavirus vaccine is proposed to provide broad protective immunity against major coronaviruses and is durable. Designing vaccines that activate T-cells is yet another route toward a pan-coronavirus vaccine. T-cells and inflammatory cytokines may contribute to viral clearance [38]. In the present study, we used the conserved T-cell epitopes with high antigenicity values. In addition, these epitopes were present in both the S1 and S2 regions of the spike protein. However, most of the epitopes were located in the S2 region. The S2 region is more conserved among coronaviruses than S1. Therefore, it may elicit more cross-reactivity and thus be able to combat other betacoronaviruses, elicit a stronger and longer-lasting memory response, and reduce the likelihood of variations. Among the selected epitopes for designing a vaccine construct, few predicted epitopes have been experimentally validated. For example, epitopes T3, L2, L3, T12–T18 are reported to be present in the antigen region [39]. Similarly, the epitopes T2–T3 and L3 were also present in the linear epitope “hot” regions reported by Li et al. [40]. In light of these experimentally validated epitopes, our results and the designed methodology were further authenticated. The designed vaccine also possesses suitable physiochemical and structural properties. Interestingly, our vaccine construct is expected to strongly interact with TLR3 to enhance immune responses.

Upon multistage in silico analysis, this vaccine candidate is anticipated to strongly elicit both humoral and cell-mediated immune responses. Thus, we postulate that this vaccine could be a potentially powerful prophylactic and therapeutic agent against SARS-CoV-2. As it is designed based on the most conserved regions of the spike, it should be applicable for the current and future epidemics evolved from these related coronaviruses. Future experimental research is warranted to develop this vaccine further into real-world applications.

## Figures and Tables

**Table 1:** A list of the final selected epitopes that fulfilled all of the criteria for antigenicity (>4), nonallergenicity, non-toxicity, and could also induce the IFN-γ immune response. The respective position represents the location of epitope in the spike protein. Epitopes T1–T20 represent the MHC I epitopes while epitopes L1–L10 represent the MHCII epitopes.

Position	Epitope	Antigenicity	Name	Position	Epitope	Antigenicity	Name
**MHCI**				**MHCII**			
**316**	SNFRVQPTE	1.5	T1	512	VLSFELLHA	1	L1
**545**	GLTGTGVLT	1	T2	753	LLQYGSFCT	0.8	L2
**612**	YQDVNCTEV	1.6	T3	822	LFNKVTLAD	0.6	L3
**826**	VTLADAGFI	1.2	T4	906	FNGIGVTQN	1.2	L4
**901**	QMAYRFNGI	0.68	T5	1013	IRAAEIRAS	0.5	L5
**913**	QNVLYENQK	0.52	T6	1033	VLGQSKRVD	1.33	L6
**965**	QLSSNFGAI	0.8	T7	1060	VVFLHVTYV	1.5	L7
**1016**	AEIRASANL	0.7	T8	1061	VFLHVTYVP	1.23	L8
**1037**	SKRVDFCGK	1.7	T9	1065	VTYVPAQEK	0.8	L9
**1063**	LHVTYVPAQ	1.35	T10	1128	VVIGIVNNT	1.3	L10
**1128**	VVIGIVNNT	1.3	T11				
**1158**	NHTSPDVDL	0.84	T12				
**1181**	KEIDRLNEV	0.55	T13				
**1198**	IDLQELGKY	0.77	T14				
**1201**	QELGKYEQY	0.5	T15				
**1202**	ELGKYEQYI	0.5	T16				
**1207**	EQYIKWPWY	1.1	T17				
**1210**	IKWPWYIWL	0.9	T18				
**1257**	DEDDSEPVL	0.5	T19				
**1264**	VLKGVKLHY	1.2	T20				

## Data Availability

Not applicable.

## Supplementary Materials

The following supporting information can be downloaded at: https://www.mdpi.com/article/10.3390/vaccines10071150/s1, Table S1. All 27 conserved regions observed in SARS-CoV-2, bat SL-CoV and SARS-CoV consensus sequences of spike region; Figure S1. Illustration of generating spike consensus sequences using SARS-CoV-2 and bat SL-CoV as examples; Figure S2. Schematic representation of secondary structure prediction of spike of SARS-CoV-2 (left), bat SL-CoV (middle) and SARS-CoV (right) based on the consensus sequences; Figure S3. Schematic representation of secondary structure prediction of the multi-epitope vaccine construct.
