# Supplementary material for: Human Coronavirus Spike Protein Based Multi-Epitope Vaccine against COVID-19 and Potential Future Zoonotic Coronaviruses by Using Immunoinformatic Approaches
Source: Vaccines (Basel). 2022 Jul 19;10(7):1150. doi: 10.3390/vaccines10071150 (PMC9323133; doi:10.3390/vaccines10071150)
Supplement: Supplementary file 1 [file vaccines-10-01150-s001.zip › vaccines-1793552-supplementary.pdf]

Table S1. All 27 conserved regions observed in SARS-CoV-2, bat SL-CoV and SARS-CoV consensus sequences of spike region.

No	Conserved regions	Positions
**1**	ENGTITDAVDC	286-296
**2**	KGIYQTSNFRV	315-325
**3**	VRFPNITNLCPFGEVFNAT	332-350
**4**	ISNCVADYSVLYNS	363-376
**5**	FSTFKCYGVS	379-388
**6**	VRQIAPGQTG	412-421
**7**	IADYNYKLPDDF	423-434
**8**	GYQPYRVVVLSFELL	509-523
**9**	CVNFNFNGLTGTGVLT	543-558
**10**	PCSFGGVSVITPGTN	594-608
**11**	VAVLYQDVNCT	613-623
**12**	AGCLIGAEHV	652-661
**13**	SYECDIPIGAGICASY	664-679
**14**	MYICGDSTEC	745-754
**15**	NLLLQYGSFCTQLNRAL	756-772
**16**	FGGFNFSQILPDP	802-814
**17**	KRSFIEDLLFNKVTLADAGF	819-838
**18**	ARDLICAQKFNGLTVLPPLLTD	851-872
**19**	GWTFGAGAALQIPFAMQMAYRFNGIGVTQNVLYENQK	890-926
**20**	ALGKLQDVVNQNAQALNTLVKQLSSNFGAISSVLNDILSRLDKVEAEVQIDRLITGRLQSLQTYVTQQLIR AAEIRASANLAATKMSECVLGQSKRVDFCGKGYHLMSFPQ	949-1059
**21**	APHGVVFLHVTYVP	1061-1074
**22**	NFTTAPAICH	1079-1088
**23**	PQIITTDNTFVSGNCDVVIGI	1117-1137
**24**	NNTVYDPLQPELDSFKEELDKYFKNHTSPDVDLGDISGINASVVNIQKEIDRLNEVAKNLNESLIDLQELGK YEQYIKWPWY	1139-1220
**25**	WLGFIAGLIAIVMVTI	1222-1237
**26**	LCCMTSCCSCLKG	1239-1251
**27**	CSCGSCCKFDEDDSEPVLKGVKLHYT	1253-1278

Figure S1. Illustration of generating spike consensus sequences using SARS-CoV-2 and bat SL-CoV as examples.

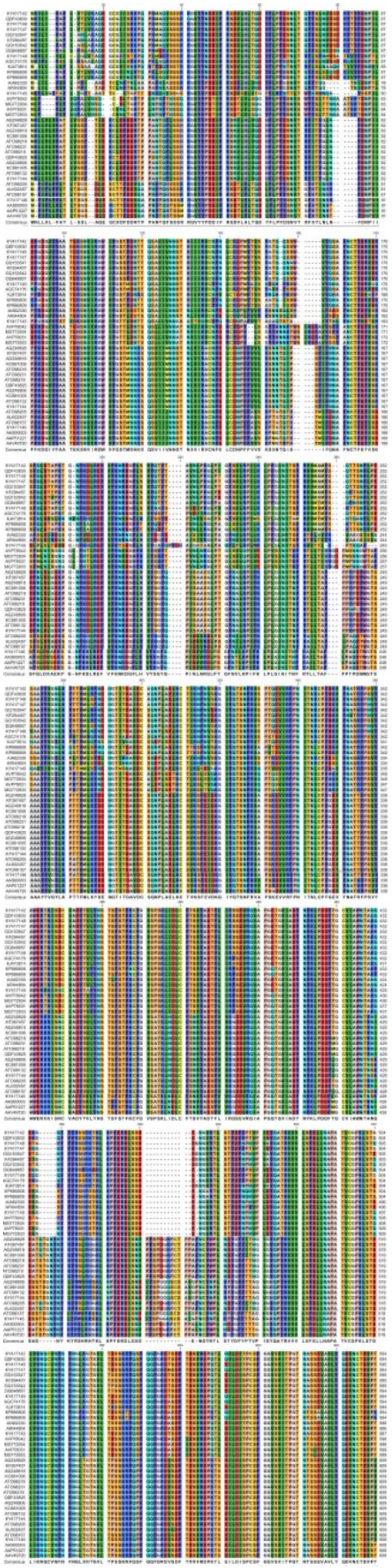

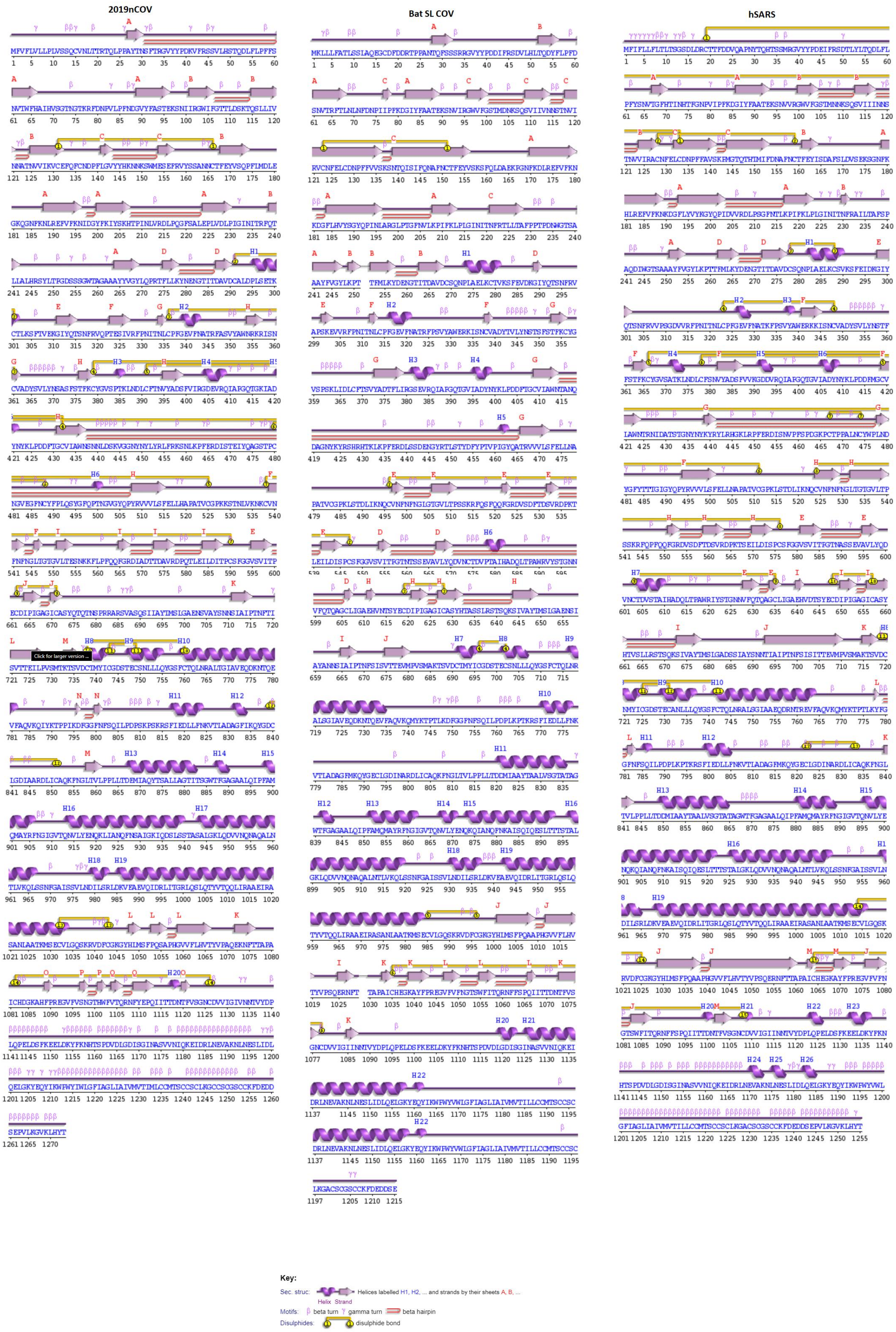
2019nCOV
Bat SL COV
hSARS
Click for larger version ...
Key:
Sec. struc: Helices labelled H1, H2, ... and strands by their sheets A, B, ...
Helix Strand
Motifs: β beta turn γ gamma turn beta hairpin
Disulphides: disulphide bond

Figure S2. Schematic representation of secondary structure prediction of spike of SARS-CoV-2 (left), bat SL-CoV (middle) and SARS-CoV (right) based on the consensus sequences. The secondary structure of SARS-CoV-2 spike contains 17 sheets, 18 beta hair pins, 18 beta bulges, 53 strands, 20 helices, 23 helix-helix nteracts, 209 beta turns, 73 gamma turns and 14 disulphides. Bat SL-CoV spike contains 12 sheets, 1 beta alpha beta unit, 18 beta hair pins, 15 beta bulges, 48 strands, 22 helices, 22 helix-helix interacts, 106 beta turns, 15 gamma turns and 6 disulphides. SARS-CoV spike contains 17 sheets, 18 beta hairpins, 19 beta buldges, 48 strands, 26 helices, 20 helix-helix interacts, 183 beta turns, 60 gamma turns, 14 disulphides.

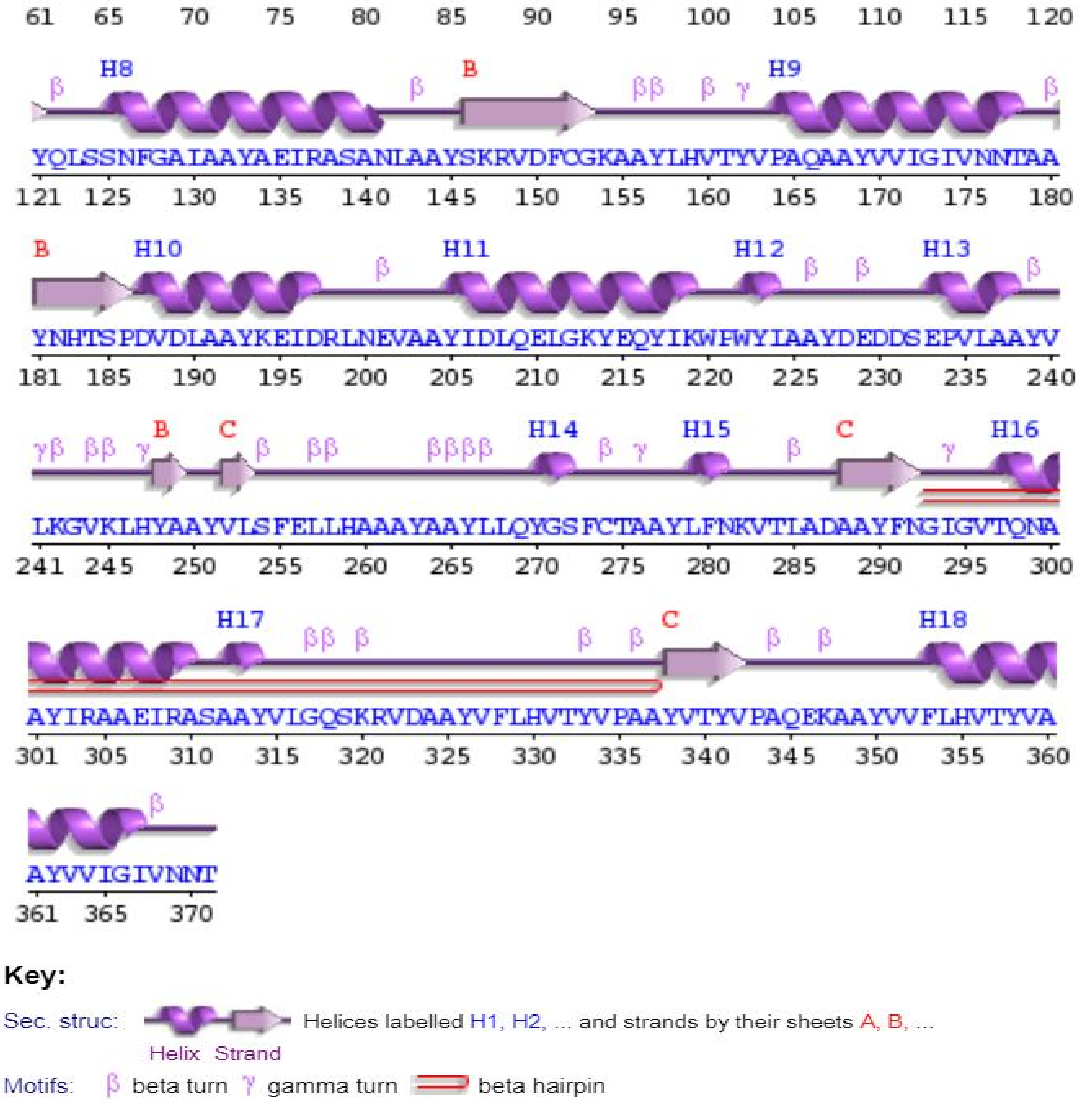

Figure S3. Schematic representation of secondary structure prediction of the multi-epitope vaccine construct. It possesses three sheets, three beta alpha beta units, two beta hairpin, one beta buldge, nine strands, 18 helices, 14 helix-helix interacts, 37 beta turn, 13 gamma turns
